# Personalized vestibular rehabilitation: medical chart survey with patients seen at the ambulatory of otoneurology of I.S.C.M.S.P.

**DOI:** 10.1016/S1808-8694(15)31196-4

**Published:** 2015-10-20

**Authors:** Lucia Kazuko Nishino, Cristina de Freitas Ganança, Andrea Manso, Carlos Alberto Herrerias de Campos, Gustavo P. Korn

**Affiliations:** 1 Specialist in Clinical Audiology, Speech and Hearing Therapist.; 2 Ph.D. studies in Communication Disorders under course, Universidade Federal de Sao Paulo - Escola Paulista de Medicina, Speech and Hearing Therapist.; 3 Specialist in Clinical Audiology.; 4 Ph.D. in Otorhinolaryngology.; 5 Master studies in Medicine under course, Program of Post-graduation in Otorhinolaryngology, Universidade Federal de Sao Paulo - Escola Paulista de Medicina, Otorhinolaryngologist.

**Keywords:** vestibular rehabilitation, personalized vestibular rehabilitation

## Abstract

The objective of this research study was to verify the efficiency of the personalized vestibular rehabilitation (PVR) in different otoneurologic clinical diseases, as well as set the best protocol option in each case. **Study design:** clinical retrospective. **Material and method:** A retrospective study was conducted based on the description of the vestibular rehabilitation program of 37 patients aged 21 to 87 years, twenty-six females and eleven males, with different clinical diseases seen in the Otoneurologic Ambulatory of Otolaryngology, department of Irmandade da Santa Casa de Misericórdia de Sao Paulo, from 2002 to 2003. Those patients went through otoneurologic evaluation and after diagnosis they were referred to vestibular rehabilitation. Each patient followed a specific program based on diagnosis, clinical disease and symptoms. We performed an individual analysis of the evaluation of each patient and group analysis in order to verify the efficiency of the PVR. **Conclusion:** It was possible to conclude that the PVR program is an effective resource in the treatment of otoneurologic symptoms of patients, consequently improving their quality of life.

## INTRODUCTION

Maintenance of body balance during body and head movement depends on the harmonious interaction of the motor sensorial systems and precise processing of information. For this reason, the Central Nervous System (CNS) needs redundant information coming from vestibular, visual and proprioceptive systems about what happens in the environment to maintain our body erect and in balance ^1^. If there is damage to one of these systems, there will be discrepancy of information leading to conflict, which may lead to onset of body imbalance symptoms, such as dizziness, which is defined as a mistaken sensation of body and environmental movement. There are different types of dizziness, which may be divided into rotation dizziness, known as vertigo and non-rotation dizziness, characterized by instability, fluctuation, sensation of fall, gait deviation, empty-head sensation, among others 1, 2, 3.

The CNS processes this information by combining it and generates responses by reflexes that improve posture control and body orientation in relation to the space, allowing the subject to make his/her daily activities. The most important reflexes are Vestibule-Ocular Reflex (RVO) and Vestibule-Spinal Reflex (RVE); RVO allows stabilization of sight during head movement and RVE generates body movement of compensation to maintain the stability of the head and body, so as to prevent falls ^1^.

Dizziness has high incidence in the world population, reported by many authors as the main complaint after the age of 65 years, present in about 80% of the population. As to origin, in 85% of the cases it is located in the vestibular system and the other 15% originates exclusively from ocular, neurological, psychiatric, metabolic and cardiovascular origin 2, 4, 5.

There is a high number of possible causes for dizziness and there are approximately 300 otoneurological clinical presentations with different clinical manifestations. This variety may be explained by the structure and physiology of the labyrinth, both in the vestibular and the auditory portion, which is very sensitive to physiological affections located in other body parts and frequently the etiological agents of vestibular dysfunctions are represented by other distant affection^2^, ^3^, ^6^, ^7^.

Owing to etiological variability, it is paramount to recognize and characterize vestibular dysfunction using a semiotic exploration test of auditory and vestibular symptoms. The success of otoneurological therapy depends on the precision of the syndromic, topographic and etiological diagnosis. The maximization of the information with the use of computers has improved the topographic diagnosis of auditory and vestibular damage; this progress of otoneurological diagnosis has resulted in optimization of resources to control acute and chronic vertigo of vestibular origin 2, 8.

The combination of therapeutic tactics, the integrated otoneurological strategy that gathers the combination of the available clinical resources recommended to each patient, produces better results that have fewer recurrences. When treatment is exclusively etiological it may not be enough for the favorable progression of the vertigo patient, and it takes a more comprehensive therapeutic approach, because only 17% of the cases reach spontaneous resolution, whereas 85% of the patients after appropriate treatment with drugs, nutritional orientation, modification of habits to correct food errors and personalized vestibular rehabilitation reach cure or significant improvement of symptoms^8^.

Vestibular rehabilitation (VR) is a highly accepted method of otoneurological treatment in the international literature, because favorable results have been evidenced in many studies. It is based on specific and repetitive physical exercises that activate neural plasticity mechanisms of the CNS, trying to reach vestibular compensation so that the subject may perform as perfectly as possible daily activities that he/she used to perform before dizziness^4^.

If there is vestibular lesion, the CNS naturally affects the functional recovery of body imbalance, through neuroplasticity. This adaptative mechanism of vestibular motor behavior is called Vestibular Compensation. In addition to this mechanism, there is also Adaptation, Habituation and Substitution, mechanisms explored in VR 2, 9.

In Vestibular Adaptation, the vestibular system learns how to receive and process information, even if inappropriate and incomplete, adapting it to the presented stimuli. Vestibular habituation is the correction or reduction of inappropriate answers when the vestibular system is stimulated and the body starts to respond accordingly. Vestibular Substitution is the key prioritization of sensorial perception that aims at replacing information related to body balance that is absent or conflicting ^2^.

However, in most cases, recovery of the adaptative mechanism of vestibular motor behavior is incomplete, and it is necessary to intervene with specific exercises, repetitive and prolonged, to maximize neuroplasticity of CNS, reached by Vestibular Rehabilitation ^2^.

Many studies that use these exercises have shown that there is considerable variability between subjects about adaptative mechanisms that are used to the compensation. Thus, the same type of vestibular damage in different subjects may have different responses in treatment and the use of one single protocol in all cases may not produce the expected efficacy 4, 9.

Many different authors showed that VR, if personalized, taking into account the patients’ complaints, clinical presentation and vestibular exam findings have better results in relation to generalized VR ^9^.

The purpose of the present study was to check the efficacy of personalized vestibular rehabilitation in patients with different symptoms and otoneurological clinical presentations seen at Irmandade da Santa Casa de Misericórdia de Sao Paulo, between May 2002 and December 2003.

## MATERIAL AND METHOD

To carry out the present study, we made medical chart analysis of 37 patients that had concluded the program of VR at the Ambulatory of Otoneurology, Irmandade Santa Casa Misericórdia de Sao Paulo (ISCMSP), between May 2002 and December 2003, of both genders and aged 21 to 87 years. We excluded all patients that until the end of the survey had not finished the etiological diagnosis with the ENT physician.

The patients were referred to the VR program by the Otorhinolaryngologist after careful collection of history and indication to the program.

The VR program was specifically designed to each patient, based on a series of specific exercises, depending on the complaint and symptoms referred by the subject, in addition to observation of diagnostic hypothesis and findings of the vestibulometry conducted with Digital Vectonystagmography, which recorded in 3 channels the ocular movement in the different vestibular-oculomotor tests: calibration of ocular movements, spontaneous nystagmus, semi-spontaneous nystagmus, saccadic ocular movement, pendulum test, optokinetic test, decreasing pendular rotation test and caloric test with air. In addition to these records, the Vestibular Exam also included Brandt-Daroff maneuver, to detect benign paroxysmal posture vertigo (VPPB) in all patients.

The device used to carry out the tests was the digital vectonystagmographer by Neurograff Eletromedicina Ltda., using software VEC WIN, with air otocalorimeter NGR-05, by the same company.

In the vestibular assessment, we found the following results of Vestibular Exam: Normal Vestibular Exam, Irritative and Deficit Peripheral Vestibular Syndrome, and Central Syndrome.

All patients submitted to VR were followed up weekly in the Ambulatory of Otoneurology, to check improvement or not of symptoms and exercises for probable change in the proposed series as patients mastered them, in addition to continuous verification of sensorial conflict caused by the exercises so as to provide a progressive and effective stimulation of the vestibular system. Thus, most of these patients were submitted to more than one protocol, practicing different exercises during treatment.

Patients with chronic vertigo were instructed to make a series of exercises at home from 2 to 3 times a day, before the meals. Some of the movements required specific material, such as a mat or pillow, word card or figure, but they could be adapted to the reality of each patient.

Exercises were based on the following protocols:

1. Exercises by Cawthorne (1944) & Cooksey (1945)
  - Indicated for unilateral vestibular dysfunctions or head trauma, they prioritize tracking ocular movement, head movements in different directions, trunk and head movements, walking, climbing up or down stairs and ramps, with both open and closed eyes.
2. Herdman Protocol - Exercises to increment vestibular adaptation (Herdman, 1990 and 1996):
  - Indicated to hypofunction unilateral to vestibular stimulation.
  - Vestibular-visual interaction.
  - Increase RVO gain and tolerance to head movements.
3. Herdman Protocol - Exercises to increase stabilization of static and dynamic posture (Herdman, 1990 and 1996):
  - Indicated to unilateral or bilateral vestibular hypofunction.
  - It produces strategies to make daily life activities, even in case of visual, somatosensorial or vestibular information deprivation; it supports the development of self-confidence in patients and defines functional limits because they manipulate visual, somatosensorial and vestibular clues to force the subject to integrate and use vestibular information to maintain posture stability.
4. Herdman Exercise Protocol to stabilize eye movement (Herdman, 1990 and 1996):
  - Indicated to bilateral peripheral deficit.
  - Maximize cervical-ocular reflex and the residual vestibular-ocular function.
5. Ganança Protocol - Exercises to stimulate the optovestibular function (Ganança et al., 1989):
  - Indicated for dizziness of vestibular origin that do not benefit from other types of exercises.
6. Exercise Protocol by the Associazione Otologi Ospedalieri Italian (Bologna, 1983):
  - Indicated to chronic vertigo of peripheral origin.
  - The basic protocol contains 17 exercises.
7. Exercises by Davis & O’Leary (1995):
  - Indicated in patients with changes in gain, phase and/or symmetry of reflexes in the head rotation tests.
8. Exercise of positional vertigo:
  - Brandt-Daroff Exercises;
  - Dix-Hallpike Maneuver;
  - Epley canalicular repositioning;
  - Semont releasing maneuver.

In the final analysis of data, we took into consideration the referred complaint of each patient and the diagnostic hypothesis and findings in the vestibular exam so as to compare the results of VR in which we described the protocols used and each exercise performed. Findings were considered individually and as a whole, in an attempt to observe the efficacy of this therapeutic method in patients seen in the Institution.

Protocols were initially designed according to patients’ complaints, employing the ones that provided more sensorial conflict, and in many situations they were modified aiming at meeting the needs of each case. These variations were made to increment the exercises, such as for example to make them with closed eyes, even when the author had only described opened eyes, or modifying the surface, such as walking over pillows.

During the treatment, exercises were adapted to respect the functional limitations of each patients, such as back problems, obesity, arthrosis, among others.

The exercises were also modified if they were extremely easy for the patients to perform them, of if they did not provide sensorial conflict. These modifications were progressive and readapted in each session.

The progression was made weekly, according to the efficacy of the performance of exercises and patients’ report, taking into account the daily needs of each case.

The survey was appreciated and approved by the Research Ethics Committee of ISCMSP under nº 242/03.

## RESULTS

Using the analysis of results obtained with the Vestibular Rehabilitation of 37 patients seen in the Ambulatory of Otoneurology, I.S.C.M.S.P., we could observe the following findings:

### Characterization of the Studied Population

**Figure 1:**
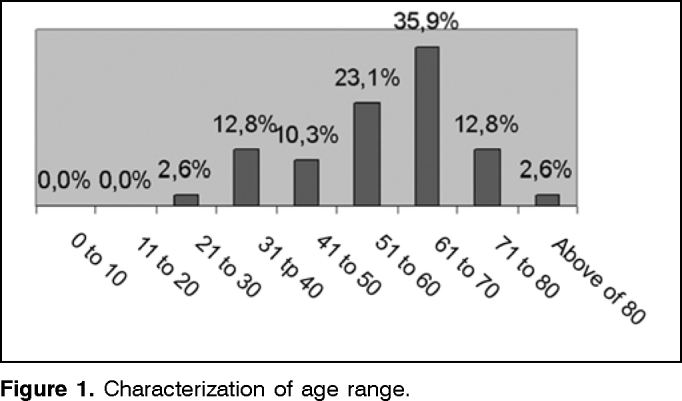
Characterization of age range.

Among the assessed cases, we found 70.27% of female and 29.73% of male subjects, ages ranging from 21 and 87 years and distributed in age ranges (Figure 1).

### Characterization of the Results of Vestibular Exam

**Figure 4:**
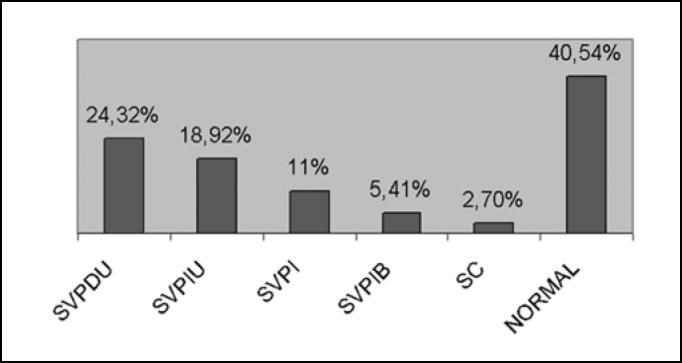
Characterization of the result of the vestibular exam. Key: SVPDU: Unilateral Deficit Peripheral Vestibular Syndrome /SVPIU = Unilateral Irritative Peripheral Vestibular Syndrome/ SVPI = Irritative Peripheral Vestibular Syndrome / SVPIB= Bilateral Irritative Peripheral Vestibular Syndrome/SC = Central Syndrome / NORMAL= Normal Vestibular Exam

The vestibular exam was normal in 40.54% and in 59.46% it was abnormal (Figure 4).

As to results obtained with VR, we will describe the different variables isolated and later compare them.

As to the protocols used, we observed that Epley Maneuver was performed in most of the patients - 21 patients (56.76%) that presented rotation nystagmus or vertigo in Brandt-Daroff maneuver, even with different diagnostic hypothesis, but with posture vertigo characteristics. Out of the total, 18 (48.65%) had diagnostic hypothesis of pure VPPB, 3 patients (8.10%) had post-trauma vertigo and VPPB, 1 patient (2.70%) had Ménière disease and 1 patient (2.70%) had diagnosis of vestibular Schwannoma.

The second most used protocol was exercise for stimulation of vestibular-ocular reflex, by Herdman, in 54.05% of the cases, followed by exercises for static and dynamic stimulation by Herdman (PED), gait exercises in Herdman protocol, Brandt-Daroff maneuver, Associazione Otologi Ospedalieri Italian (AOOI) protocol, exercises of cervical-ocular reflex (RCO) stimulation, exercise with optokinetic stimulation by Ganança et al. (OPTO) and Semont maneuver (Table 1).

**Table 1:** Protocols used in Personalized Vestibular Rehabilitation.

	Epley	BD	Semont	Gait	Protocols RCO	PED	RVO	OPTO	AOOI
total (37)	22	12	1	14	7	15	20	5	9
%	59,46	32,43	2,70	37,84	18,92	40,54	54,05	13,51	24,32
VPPB (23)	22	7	1	2	1	3	5	-	3
%	91,67	29,17	4,17	8,33	4,17	12,50	20,83	-	12,50
MÉNIÈRE (6)	1	4	-	4	3	3	6	1	4
%	16,67	66,67	-	66,67	50,00	50,00	100,00	16,67	66,67
VEST.VASCULAR (2)	-	-	-	2	-	2	2	-	1
%	-	-	-	100,00	-	100,00	100,00	-	50,00
VEST.METABOLIC (2)	-	1	-	2	1	2	2	2	-
%	-	50,00	-	100,00	50,00	100,00	100,00	100,00	-
SUDDEN DEAFN (1)	-	-	-	1		1	1	-	1
%	-	-	-	100,00	100,00	100,00	100,00	-	100,00
V.POST-TRAUMA (5)	3	2	1	3	3	3	3	1	1
%	60,00	40,00	20,00	60,00	60,00	60,00	60,00	20,00	20,00
T.CEREBELLAR (1)	-	-	1	-	1	1	-	-	
%	-	-	-	-	-	100,00	-	-	-
VESTIBULAR SCHWANNOMA (1)	-	-	-	-	-	-	-	-	-
%	-	-	-	-	-	-	100,00	-	-

Key: VPPB= benign paroxysmal postural vertigo/ Ménière = Ménière disease/ VEST. = vestibulopathy v. post-trauma = post-trauma vertigo / T.= tumor

The total number of sessions ranged from 1 to 15, and it was directly related with number of protocols used by patient, that is, the patients with longer time in therapy were submitted to more exercises from different protocols. Patients with VPPB were discharged earlier than other patients with chronic vertigo.

Upon discharge, 91.89% (35) had significant improvement of symptoms and 8.10% (3) were referred to other treatments.

## DISCUSSION

In view of the objective of the present study, which tried to show the results of personalized Vestibular Rehabilitation in patients with different otoneurological symptoms and clinical presentations, we could observe higher prevalence in female cases, approximately twice higher, a proportion that had already been detected by other authors in patients with dizziness 5, 10.

As to age range, we noticed higher prevalence in ages of 61 and 70 years (35.9%), from 51 to 60 years (23.1%), from 31 to 40 years and from 71 to 80 years (12.8%), and those results are in agreement with Ganança et al.^10^, who studied one thousand consecutive otoneurological patients and found prevalence mainly in adults and elderly cases.

We found many different diagnostic hypothesis, and VPPB is the most common one, followed by Ménière disease and post-trauma vertigo. According to Ganança et al.^10^, VPPB and Ménière disease are the two most frequent labyrinthopathologies, because they occur mainly in adult life.

**Figure 2:**
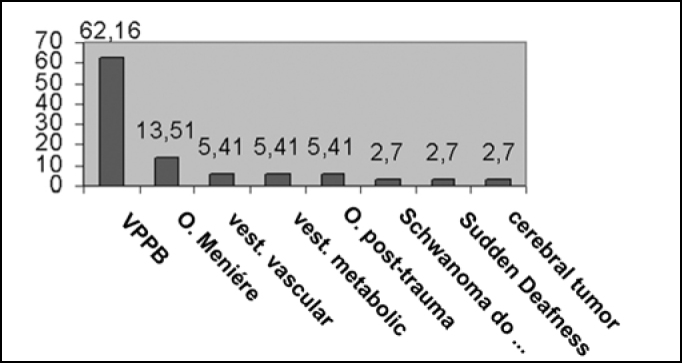
Characterization of diagnostic hypotheses (value in percentage).

**Figure 3:**
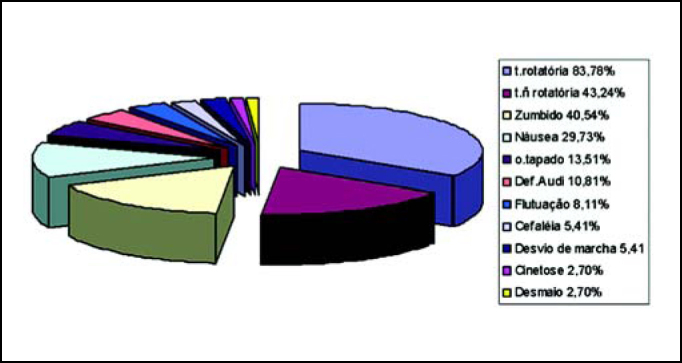
Characterization of otoneurological symptoms referred by patients.

Key: t.rotatória= rotation dizziness / t.ñ= non-rotation dizziness/ tapado= ear fullness sensation / Def.Audi = hearing loss

**Table 2:** Relation of number of sessions x protocols used.

N	Diagnostic Hypothesis	Sessions	Vestibular Exam	Protocols						
4	VPPB	1	Ny Unilateral	Normal	Epley					
1	VPPB	5	Ny Unilateral	Normal	Epley	PED	RVO	AOOI		
1	VPPB	1	Ny Unilateral	SVPI	Epley					
2	VPPB	1	Ny Unilateral	SVPIU	Epley					
2	VPPB	4	Ny Unilateral	SVPIU	Epley					
1	VPPB	2	Ny Unilateral	SVPDU	Epley					
1	VPPB	5	Ny Unilateral	SVPDU	Epley	Brandt Daroff				
1	VPPB	3	Ny bilateral	Normal	Epley					
1	VPPB	9	Ny bilateral	Normal	Epley	Brandt Daroff				
1	VPPB	5	Ny bilateral	SVPIU	Epley	Brandt Daroff				
1	VPPB	3		SVPDU	RVO					
1	VPPB	6		SVPIU	Epley	RVO	Brandt Daroff	PED	Gait	AOOI
1	VPPB	4		Normal	Epley	RVO	Brandt Daroff			
1	V. POST-TRAUMA/VPPB	3		Normal	Epley	Brandt Daroff				
1	V. POST-TRAUMA/VPPB	10	Ny Unilateral	Normal	Epley	Brandt Daroff	Semont	RCO	AOOI	
1	V. POST-TRAUMA/VPPB	11	Ny Unilateral	SVPDU	Epley	RVO	PED	AOOI		
1	V. POST-TRAUMA	7		SVPDU	Gait	RVO	PED	RCO		
1	V. POST-TRAUMA	15		SVPI	Gait	RVO	PED	Opto	RCO	
1	MÉNIÈRE	3		Normal	Brandt Daroff	RVO	AOOI			
1	MÉNIÈRE	14		SVPIU	Brandt Daroff	RVO	PED	AOOI	Gait	OPTO
1	MÉNIÈRE	6		SVPDU	Gait	RVO	RCO	AOOI	PED	
1	MÉNIÈRE	4		SVPDU	Gait	RVO	RCO			
1	MÉNIÈRE	8		SVPDU	Brandt Daroff	Gait	RVO	RCO	AOOI	PED
1	MÉNIÈRE/VPPB	4	Ny Unilateral	SVPDU	Gait	RVO				
1	SUDDEN DEAFNESS	3		SVPIB	Gait	RVO	PED	AOOI		
1	Schwannoma/IVB/VPPB	4	Ny Unilateral	Normal	Epley					
1	CEREBELLAR TUMOR	4		SVPI/Central	Gait	PED	RVO			
1	VEST. VASCULAR	4		SVPI	Gait	RVO	PED			
1	VEST. VASCULAR	4		Normal	Gait	PED	RVO	AOOI		
1	VESTIBULAR NEURITIS	7		SVPI	RVO	Opto				
1	VEST. METABOLIC	8		Normal	Gait	RCO	RVO	PED	OPTO	
1	VEST. METABOLIC	9		SVPIB	Gait	RCO	RVO	PED	AOOI	

Key: VPPB= benign paroxysmal postural vertigo/ Ménière = Ménière disease/ VEST. = vestibulopathy

IVB= vertebrobasilar insufficiency

Ny= nystagmus/: SVPDU = Unilateral Deficit Peripheral Vestibular Syndrome /SVPIU = Unilateral Irritative Peripheral Vestibular Syndrome/ SVPI = Irritative Peripheral Vestibular Syndrome / SVPIB= Bilateral Irritative Peripheral Vestibular Syndrome

SC = Central Syndrome / NORMAL= Normal Vestibular Exam

RVO= vestibule-ocular reflex (Davis & O’Leary Protocol)/ PED= Herdman protocol - static-dynamic posture / RCO= cervical-ocular reflex (Herdman protocol - sight stabilization/OPTO=optokinetic (Ganança Protocol)/AOOI = Associazione Otologi Ospedalieri Italian

As to the most frequently reported otoneurological symptoms, rotation dizziness was the most common one, present in 83.78% of the cases, followed by non-rotation dizziness (43.24%), tinnitus (40.54%) and nausea (29.73%). Caovilla et al.^11^ also found dizziness (rotation and non-rotation dizziness) as the most common symptom (100%), followed by tinnitus (24.9%).

The Vestibular rehabilitation applied in our patients had a personalized approach to restore body balance. In many cases, it suffered modifications in relation to the advocated exercises and we made changes to conflict stability so as to provide a set of progression that respected difficulties and skills that patients had in each exercise. Angeli et al.^12^ also combined other protocols in the vestibular rehabilitation taking into account the patients’ complaints and reaching the best results in treating VPPB. Toledo et al.^13^ also used Semont maneuver for VPPB, detecting high recurrence of symptoms when performed isolated, which did not occur with the repositioning maneuver combined with other exercises, reaching 100% of cure in the patients.

O’Reilly et al.^14^ stated that canalicular reposition has shown high efficiency in all forms of VPPB, but there were some patients that required additional intervention to exclude all symptoms, mainly secondary VPPB. Banfield et al.^15^ noticed excellent short-term results but they had high recurrence of symptoms when using isolated Epley maneuver. Bittar et al.^16^ noticed that some patients did not satisfactorily respond to vestibular rehabilitation, even when they performed the recommended exercises. There were 116 patients that followed one single protocol, Cawthorne & Cooksey protocol, which made them reflect about the need to use other existing protocols to satisfactorily increase responses to treatment. We could detect, thus, that the authors who used only the protocol as treatment approach were less successful than those that combined other techniques to the protocol.

Out of the cases seen by us, we detected that the number of sessions varied depending on the amount of protocols used and the association with clinical presentations, location of lesion and nature of the symptom. However, despite influencing the extension of treatment, it did not hinder the results, which were quite satisfactory as shown by other authors ^27^.

Upon discharge, 91.89% (35) had improved significantly their symptoms and 8.10% (3) had not responded to treatment. Out of these patients, we detected associated psychological or psychiatric condition, and referred them to other therapeutic methods or reviewed the etiological and syndromic presentations. Until the end of the follow up, they were still waiting for specialized treatment and then carry on the program under appropriate guidance. We proceeded as such because according to Bittar et al.^16^, patients that have psychiatric problems are difficult to treat because dizziness does not result from a specific lesion, but rather from a complex set of neurological and behavioral affections, in which only the appropriate psychiatric treatment will lead to the desired effect.

## CONCLUSION

According to the results of the personalized Vestibular Rehabilitation in 37 patients, we detected efficiency of treatment for different otoneurological clinical presentations. We observed that the use of different protocols increased efficacy of treatment and consequent elimination or attenuation of symptoms in most vertigo patients.

Moreover, we observed that in some cases, even in patients with diagnostic hypothesis and similar vestibular exam, different number of sessions and types of protocols were required. It showed us the importance of a personalized rehabilitation program respecting not only the lesion site, but also patients’ complaints, difficulties and skills.

The therapeutic success made us believe in Personalized Vestibular Rehabilitation as an excellent therapeutic option, because in addition to showing significant improvement in body balance, it also increased the self-confidence lost by the patients, with direct improvement in quality of life.
